# The Role of Artistic Creative Activities in Navigating the COVID-19 Pandemic in Australia

**DOI:** 10.3389/fpsyg.2021.696202

**Published:** 2021-08-25

**Authors:** Frederic Kiernan, Anthony Chmiel, Sandra Garrido, Martha Hickey, Jane W. Davidson

**Affiliations:** ^1^Melbourne Conservatorium of Music, University of Melbourne, Melbourne, VIC, Australia; ^2^The MARCS Institute for Brain, Behaviour, and Development, Western Sydney University, Sydney, NSW, Australia; ^3^Department of Obstetrics and Gynaecology, University of Melbourne, Melbourne, VIC, Australia; ^4^Royal Women's Hospital, Melbourne, VIC, Australia

**Keywords:** artistic creative activities, COVID-19, coronavirus, creativity, emotion regulation, musical engagement, well-being, mental health

## Abstract

During the COVID-19 pandemic some Australians turned to artistic creative activities (ACAs) as a way of managing their own mental health and well-being. This study examined the role of ACAs in regulating emotion and supporting mental health and well-being during the COVID-19 pandemic, and also attempted to identify at-risk populations. We proposed that (1) participants would use ACAs as avoidance-based emotion regulation strategies; and (2) music engagement would be used for emotion regulation. Australian participants (*N* = 653) recruited from the general public completed an online survey, which included scales targeting anxiety (GAD7 scale), depression (PHQ9 scale) and loneliness (two UCLA Loneliness Scales, referring to “Before” and “Since” COVID-19). Participants reported which ACAs they had undertaken and ceased during the pandemic using an established list and ranked their undertaken ACAs in terms of effectiveness at making them “feel better.” For their top-ranked ACA, participants then completed the Emotion Regulation Scale for Artistic Creative Activities (ERS-ACA), and if participants had undertaken any musical ACAs, also the Musical Engagement Questionnaire (MusEQ). The results supported both hypotheses. ANOVAs indicated that participants ranked significantly higher on the “avoidance” ERS-ACA subscale than the other subscales, and that participants ranked significantly higher on the emotion regulation and musical preference MusEQ subscales than the other subscales. Additionally, while ACAs such as “Watching films or TV shows” and “Cookery or baking” were common, they ranked poorly as effective methods of emotion regulation, whereas “Listening to music” was the second-most frequently undertaken ACA and also the most effective. “Singing” and “Dancing” were among the most ceased ACAs but also ranked among the most effective for emotion regulation, suggesting that support for developing pandemic-safe approaches to these ACAs may provide well-being benefits in future crises. Additionally, correlation analyses showed that younger participants, those who took less exercise during the pandemic, and those with the highest musical engagement reported the poorest well-being. We conclude that ACAs provided an important resource for supporting mental health and well-being during the COVID-19 pandemic in Australia and could potentially support mental health and well-being in future crises.

## Introduction

The COVID-19 pandemic has taken a substantial global toll on health and well-being. As of 12 March 2021, over 117 million people had been infected with the virus with over 2.6 million deaths. These deaths and restrictions to contain the disease have had immense socio-economic implications (Nicola et al., 2020), which together place a burden on overall mental health and well-being (Fisher et al., 2020; Khan et al., 2020). During 2020, daily life changed radically for many, with lockdowns and social-distancing measures being implemented to curb infection rates (Lewnard and Lo, 2020; Warren and Bordoloi, 2020). Individuals and groups at all levels of society have had to manage the consequences of these changes. Health workers have had their duties augmented, with many working to monitor infection spread, care for those infected, and implement rigorous sanitizing and personal protection procedures to minimize their own exposure to the virus (Nienhaus and Hod, 2020). Many retail and entertainment businesses have crumbled, sought temporary government support and/or adapted their business models by offering services online (Bartik et al., 2020; Kim, 2020; Seetharaman, 2020), while other forms of employment have required workers to transfer their duties to home (Bonacini et al., 2021). The creative industries (e.g., film, advertising and fashion, as well as creative occupations such as musicians, dancers, actors, visual artists and designers) have faced immense pressure due to the rapid spread of the virus and physical distancing restrictions. Losses in these industries between 1 April and 31 July 2020 in the United States alone have been estimated at 2.7 million jobs and more than $150 billion USD in sales of goods and services, with the fine and performing arts industries being hardest hit (Florida and Seman, 2020). Many creative and performing arts organizations have also turned to online alternatives (Keller, 2020). The pandemic has thus caused a great deal of stress and fear, even for those who have not been infected (Pfefferbaum and North, 2020).

Alongside these negative impacts, the COVID-19 pandemic has also provided some positive creative opportunities for adaptation (Kapoor and Kaufman, 2020; Kirchner et al., 2021). Emerging evidence suggests that social-distancing measures and stay-at-home orders have encouraged engagement in creative arts activities (Gupta, 2020; Radermecker, 2020). This has included commonplace activities such as baking (Easterbrook-Smith, 2020) and watching films (Mikos, 2020), as well as new and emerging forms of artistic engagement such as singing in online choirs or from balconies (Taylor, 2020; see also www.musicacrossthebalconies.com) and visiting online art galleries (Shehadi, 2020). Creative and artistic activities have served a range of purposes during the COVID-19 pandemic in a variety of cultural contexts, such as health promotion, improving environmental aesthetics and memorializing (de-Graft Aikins, 2020), awareness-raising about the threats posed by the pandemic (Blanc et al., 2020), and emotion regulation (Karwowski et al., 2021). And, while musical activities can serve a range of purposes such as self-expression and social group bonding (Vanstone et al., 2016), musical activities have also facilitated emotion regulation during the pandemic (Martín et al., 2021; Steinberg et al., 2021).

This evidence suggests that creative activities, including specifically artistic creative activities (hereafter ACAs)—defined as creative activities involving the arts specifically (Fancourt et al., 2019)—have supported individuals and groups in regulating their emotions during the pandemic and in supporting their own mental health and well-being. Much of the literature on the emotional and mental health impacts of pandemics and global health crises is either descriptive (e.g., documenting negative effects) and/or prescriptive (e.g., suggesting coping strategies without testing their effectiveness) (Restubog et al., 2020). As such, studies on the role of creative activities in navigating pandemic crises contribute to broader understandings of human psychological responses to epidemic encounters, which are otherwise poorly understood (Curson, 2015).

While research related to the impact of COVID-19 on daily life emerged rapidly from countries with early and restrictive lockdowns (March–June 2020), much less is known about the Australian experience where infection and death rates have been relatively low, partly due to the early and prompt enforcement of restrictions (Pew Research Center, 2020; Hong et al., 2021; World Health Organisation, 2021); by 12 March 2021, Australia had recorded 29,090 total cases and 909 deaths (Australian Government Department of Health, 2021). Much of Australia's success in containing the spread of the virus hinged on two periods of lockdown, with the first lockdown enforced nationwide for a duration of 8 weeks from late March 2020, and the second occurring in the state of Victoria for the 8 weeks following 8 July 2020. These lockdowns in turn raised new questions about the mental health and well-being of the Australian population and the ways these could be effectively supported (Fisher et al., 2020).

There is now a rich body of literature examining the links between creativity and well-being from a range of disciplinary perspectives (Humes, 2011; Gillam, 2018; Garcês et al., 2020; Kiernan et al., 2020). Psychological and philosophical approaches to the study of creativity have often focused on the “Four Ps” of creativity: people, product, process, and press (i.e., environment) (Rhodes, 1961), or have identified different levels of creative behavior, such as the “Four C model,” containing “Big-C” (eminent-level creativity), “Pro-c” (professional level creativity), “little-c” (everyday creativity) and “mini-c” (rudimentary creativity) (Kaufman and Beghetto, 2009). Such approaches have typically viewed creativity as the production of novelty that is both relevant and effective or appropriate in a given context (Cropley, 2011), and as such they usually position creative behavior in opposition to routine or habitual behavior. However, creativity and routine may be interrelated with habits forming a basis for creativity (Dalton, 2004; Chan, 2016). Some studies have also expanded the unit of analysis in creativity research by treating creativity as a socio-cultural act (Glaveanu, 2015), which may be distributed across and between multiple actors and elements (Glaveanu, 2014; Clarke and Doffman, 2017; O'Dair, 2019). Alongside these developments, creativity has also become increasingly relevant to the study of well-being (Basadur and Basadur, 2011; Krippner, 2011; Csikszentmihalyi, 2014; Gillam, 2018; Barker et al., 2019; Kiernan et al., 2020), where emotions themselves may be viewed from a sociological perspective as creative practices (Kiernan, 2020).

The term “well-being,” like “creativity,” also has multiple, domain-specific definitions (Dodge et al., 2012; Oades and Heazlewood, 2017). The hedonic tradition of well-being research has emphasized emotion-related elements such as pleasure attainment and pain avoidance and is sometimes referred to as “subjective well-being” (Kahneman et al., 1991; Diener, 2009), while the eudaimonic tradition has focused on issues such as meaning, self-realization and human development, which are typically associated with “psychological well-being” (Rogers, 1961; Keyes, 2002; Ryff and Singer, 2008; Vittersø, 2016). Well-being research has often intersected with the study of mental health (Gillam, 2018; Barker et al., 2019), partly because anxiety and depression (Galinha and Pais-Ribeiro, 2012) and loneliness (VanderWeele et al., 2012) have been shown to predict subjective well-being. Given this breadth, studies in well-being must clearly delimit which aspect of well-being is being investigated (Dodge et al., 2012). An increasing body of research is also demonstrating clear links between the creative arts, health and well-being (Clift and Camic, 2016; APPG, 2017). Recent studies have proposed that specific emotion-regulation strategies can underpin engagement in ACAs (Fancourt et al., 2019, 2020), while other studies have taken a broader approach to considering the use of ACAs in regulating emotion (in the short-term), mood (in the mid-term) and mental health (in the long-term) (Leckey, 2011; Lith et al., 2013; Fancourt et al., 2016a,b; Vanstone et al., 2016; Garrido, 2017; Leubner and Hinterberger, 2017; Krause et al., 2018; Williams et al., 2018, 2019).

### Emotion Regulation

This research suggests that ACAs may have supported the mental health and well-being of Australians during the COVID-19 pandemic via different emotion regulation strategies. Emotion regulation in the field of psychology is broadly defined as the way individuals influence which emotions they have, and when and how they experience and express these emotions (Gross, 1998). Emotion regulation may involve relatively automatic as well as more conscious behavioral attempts to modulate emotional states, including by altering one's external environment or the manner of an emotional expression (Eisenberg and Spinrad, 2004). Numerous studies have examined the links between emotion regulation and well-being across the lifespan (Joseph and Newman, 2010; Röll et al., 2012; Rice, 2015; Daniel et al., 2020; Zamani Zarchi et al., 2020), with emotion regulation typically being viewed as “prohedonic,” meaning that it is always directed at optimizing one's well-being (Riediger and Klipker, 2014). However, some studies have suggested that this may not always be true—that emotion regulation can sometimes be “contrahedonic”—since people may dwell on negative experiences in order to intensify them, or to lessen positive emotional experiences (Erber and Erber, 2000; Riediger et al., 2009).

Various explanatory frameworks for emotion regulation have been proposed. Gross' (2001) “process model” differentiates emotion regulation strategies along the timeline of the unfolding emotional response, but has been criticized for being too linear and ignoring the influence of context on emotional experience (Guendelman et al., 2017). Emotion regulation strategies have alternatively been classified as either “healthy/adaptive” or “unhealthy/maladaptive,” where the former category refers to strategies such as reappraisal, problem solving and acceptance, and the latter to strategies such as avoidance, rumination and suppression (Aldao et al., 2010). Fancourt et al. (2019) have proposed that ACAs may regulate emotion via different strategies that fall into three categories: (1) avoidance strategies such as distraction, suppression and detachment; (2) approach strategies such as acceptance, reappraisal and problem solving; and (3) self-development strategies which enhance self-identity, improve self-esteem and increase agency. Some studies have suggested that proactive, approach-oriented emotion regulation strategies are more effective at managing negative emotions in certain contexts (e.g., in workplaces) than avoidance-based strategies (Diefendorff et al., 2008; Pekaar et al., 2018), although one recent study found that avoidance-based strategies were protective of quality of life during the COVID-19 pandemic (Panayiotou et al., 2021), suggesting that such strategies can also support well-being. Research has also shown that different types of situation can inform the specific strategy deployed, with low-intensity stimuli leading to a tendency toward reappraisal strategies on the one hand, and high-intensity stimuli leading to a tendency toward distraction strategies on the other hand (Sheppes et al., 2014). Given the scale and intensity of the COVID-19 pandemic, the lack of perceived control over the spread of the virus, and its negative impacts on mental health and well-being (Fisher et al., 2020), one might reasonably expect that avoidance- distraction- or escapism-based emotion regulation strategies would be used by people seeking to support and protect their own mental health and well-being during the pandemic. Furthermore, given that creative activities have played an important role in shaping peoples' response to the pandemic, it is plausible that such emotion regulation strategies might be deployed via engagement in ACAs. And, given that previous studies have also shown that musical activities have facilitated emotion regulation during the pandemic (Martín et al., 2021; Steinberg et al., 2021), it is plausible that Australians have also used music for this purpose rather than for other reasons such as self-expression or social group bonding (Vanstone et al., 2016).

## Aims

The principal aim of this study was to investigate the role of ACAs in regulating emotion and supporting the mental health and well-being of people in Australia during the COVID-19 pandemic. The secondary aim was to identify which demographic populations were most at risk of poor mental health and well-being. Specifically, we investigated the following five research questions (RQs):

*RQ 1*: Which ACAs have people in Australia been undertaking during the COVID-19 pandemic, and how much time have they been spending on these ACAs?
*RQ 2*: Which ACAs have people in Australia ceased due to the pandemic?
*RQ 3*: Which ACAs do people in Australia report as being the most effective at making them “feel better” during the COVID-19 pandemic?
*RQ 4:* Which emotion regulation strategies have people in Australia employed during the pandemic, specifically through engagement in ACAs?
*RQ 5*: What relationships can be observed during the COVID-19 pandemic between a) engagement in ACAs, b) demographic aspects such as age, household size, and amount of exercise undertaken, and c) mental health and well-being?

Based on the literature discussed in section “Emotion regulation,” our principal hypothesis was that ACAs would be used during the pandemic to employ avoidance-based emotion regulation strategies rather than approach-based or self-development-based strategies, as defined by Fancourt et al. (2019). Our secondary hypothesis was that music would be used during the pandemic to regulate emotion.

## Method

Data were gathered for this study using an online, cross-sectional survey of people residing in Australia.

### Participants

A total of 952 participants from the general population responded to an online survey between 29 May 2020 and 16 October 2020. Of the 952 responses, 267 (28%) were incomplete and so were removed from the dataset. Furthermore, 32 responses (3.4% of the overall sample) were made from participants living outside of Australia. These responses were also removed from the dataset, in line with the research focus on well-being of Australians and to keep the sample as homogenous as possible. This led to a final sample of *N* = 653. Five-hundred and fifty-one participants (84.4%) self-identified as female, 87 (13.3%) self-identified as male, 6 (0.9%) selected “other,” and 9 (1.4%) selected “prefer not to say.” Participants also indicated which Age category they belonged to. One-hundred and twenty-seven participants (19.4%) were aged 18-24, 127 (19.4%) were aged 25-34, 144 (22%) were aged 35-44, 150 (23%) were aged 45-54, 75 (11.5%) were aged 55–64, and 30 (4.6%) were aged 65 and older.

### Measures

#### Artistic Creative Activities, Emotion Regulation, and Music Engagement

To collect data about ACAs undertaken and/or ceased during the pandemic, a list of 26 ACAs was developed (see Table 1), based on that used by Fancourt et al. (2019). Participants could also add up to four unlisted ACAs via “Other” options that were accompanied by open-ended response boxes. Fancourt et al.'s (2020) list contained 17 ACAs, although for our list some of these singular activities were split into multiple activities (e.g., “pottery, calligraphy or jewelery making” was split into three separate activities). Fancourt et al. (2019) also used the definition proposed for population-level research (Davies et al., 2012), cross-referenced with that used in the UK *Taking Part* survey (DCMS, 2017), as a basis for producing their list of ACAs, and they adopted the theoretical standpoint that ACAs are multimodal activities that combine different overlapping components but which all fulfill the same basic criteria of “art” proposed by Dutton (2006). For this reason, they did not include music listening or watching TV or films in their list of ACAs (although they did include reading). However, Davies et al. (2012) also emphasized the importance of defining arts engagement in terms of art forms and levels of engagement and identified both the active creation of art and receptive observing or listening as “artistic activities,” including music listening and watching TV/films (p. 204–214; see also Australia Council, 2010). Given that the receptive observance of art (broadly construed to include visual art, film, television, music and some digital media) can facilitate creative change by generating new perspectives on what is already familiar (Chan, 2016), and the fact that domestic media consumption typically increases the longer people stay at home (Mikos, 2020), these activities were classified as ACAs for the purposes of the current study.

**Table 1:** List of ACAs used in the survey.

**Artistic creative activities (ACAs)**
Calligraphy
Composing music/songs
Cookery or baking
Creating art/animations on computer
Creative writing
Dancing
Fashion/costume design
Gardening/attending indoor plants
Jewelery making
Learning/practicing magic tricks/circus skills
Listening to music
Make-up artistry
Making films or videos
Other
Painting or drawing
Photography
Playing a musical instrument
Playing video games
Pottery/ceramics
Reading novels, stories, poetry, or plays
Rehearsing/performing play, drama, opera, musical theater
Sculpture
Singing
Textile crafts
Viewing/contemplating artworks (books, online…)
Watching films or TV shows
Wood crafts (carving, furniture…)

*Participants were able to select up to four unlisted ACAs via “Other” options and were asked to specify these ACAs*.

Furthermore, although Fancourt et al. (2019) did not include playing video games in their list of ACAs, there is a growing body of scholarship that classifies gaming as creative and as one of the creative industries (O'Dair, 2019), and which positions gaming within discourses of aesthetic criticism (Swalwell and Wilson, 2008; Richardson et al., 2013). While Dutton (2006) also excluded video games from the category of “art,” gaming technology and aesthetics has developed significantly in the last 15 years and it can be argued that video games increasingly meet Dutton's recognition of criteria for art (Dutton, 2006, pp. 369–373). For this reason, video gaming was also included as an ACA in the current study. In summary, the survey list of ACAs used in the current study differed from Fancourt et al.'s (2019) list in that it also included the ACAs “Listening to music (live or recorded),” “Watching films, TV shows,” and “Playing video games.”

To collect data about the types of musical activities participants had been engaging in during the pandemic, if any, the 35-item Music Engagement Questionnaire was used (MusEQ; see Vanstone et al., 2016). The MusEQ scale aims to measure engagement with music in everyday life and comprises six subscales: the “Daily” subscale concerns the role of music in the routine aspects of daily life; the “Emotion” subscale relates to the emotional and mood regulatory aspects of musical experience; the “Perform” subscale concerns the social “performance” of a musical identity; the “Consume” subscale addresses consumer choices in a typical sense with regard to music; the “Respond” subscale addresses responses made in synchrony with music being heard (e.g., foot tapping or humming); and, the “Prefer” subscale addresses the degree to which the subject shows preferences or dislikes for certain styles of music (Vanstone et al., 2016).

To understand the emotion regulation strategies that underpin participants' engagement in ACAs, the 18-item “Emotion Regulation Strategies for Artistic Creative Activities Scale” was used (ERS-ACA; see Fancourt et al., 2019). This scale is split into four subscales, with the first “General Factor” being the overall *M* calculated for all 18 items, and the remaining three scales focusing on specific emotion regulation strategies. The “Avoidance” subscale was the *M* calculated for seven items that focus on emotion regulation strategies concerning distraction or escapism from negative experiences, and the “Approach” subscale was the *M* calculated for six items that focus on emotion regulation strategies concerning active confrontation of experiences. The fourth subscale, “Self-development” was the *M* calculated for the remaining five items, which focus on emotion regulation strategies concerning reaffirmation of a sense of self.

#### Well-Being Scales

Four measures pertaining to mental health and well-being were used. These were the 7-item General Anxiety Disorder Scale (GAD7; Spitzer et al., 2006), the 9-item Patient Health Questionnaire Depression scale (PHQ9; see Kroenke et al., 2001) and the Three-Item Loneliness Scale (Hughes et al., 2004), which is based on the UCLA Loneliness Scale (Russell, 1996). The Loneliness scale was administered twice, with the first time asking for responses relating to “Before the COVID-19 pandemic began” and the second time asking for responses relating to “Since the COVID-19 pandemic began.” Henceforth, these scales are referred to as “UCLA Before COVID-19” and “UCLA Since COVID-19,” respectively. These four scales were intended to give a general overview of the mental health and well-being of Australians during the COVID-19 pandemic. In this article the term “well-being scales” is used as an umbrella term for these scales, although the authors acknowledge that these scales do not cover the entire breadth of participant well-being (Dodge et al., 2012).

The GAD7 and PHQ9 scales each produce an aggregate ranging from 0 to 21 and 0 to 27, respectively, whereas the two UCLA scales each produce an aggregate ranging from 0 to 6. As these four scales were designed for the diagnosis of anxiety, depression, and loneliness, in their original format the minimum rating of “0” reflects the lowest level of anxiety, depression, or loneliness, and the highest levels of these traits are intended to be scored with the maximum possible ratings (21, 27, and 6, respectively). However, in the present paper we inverted these four scales for ease of correlations and general observations with other data. As such, GAD7 ranged from 0 (most anxiety) to 21 (least anxiety), PHQ9 ranged from 0 (most depression) to 27 (least depression) and the two UCLA scales ranged from 0 (most loneliness) to 6 (least loneliness). In other words, in the present work these four scales could be considered as ranging from feeling worst (minimum rating) to feeling best (maximum rating).

### Procedure

The survey was created with Qualtrics and distributed via an online link. The link was shared via mailing lists, websites, social media, and the like, and was completed by participants remotely on their personal devices (computer, tablet, or smartphone). Before starting the survey, all participants read through a Plain Language Statement and Consent Form. This study received approval from the Human Research Ethics Committee of the University of Melbourne (Ethics ID 2056873.1). Participation was voluntary, and the survey could be stopped at any point after commencement. The survey questions were divided into three blocks—demographics, ACAs, and well-being—which were presented to participants in randomized order.

In the ACAs block, participants were asked to indicate which ACAs, if any, they had been undertaking since the COVID-19 pandemic began. Up to four “Other” free-text options were available to select ACAs that were not specifically listed. For any ACAs that the participant selected, they were asked to enter the average amount of weekly hours they would spend on that ACA during the pandemic; the sum of all logged hours for a participant are henceforth referred to as the variable *Activity hours*. If the participant selected at least one musical ACA (singing; playing a musical instrument; composing music or songs; rehearsing or performing in a play/drama/opera/musical theater; dancing; listening to music), they were asked to complete the MusEQ scale (Vanstone et al., 2016). Participants completed the MusEQ scale only once, regardless of how many musical ACAs they selected.

Next, participants were asked to rank their selected ACAs in terms of their effectiveness in making the participant “feel better.” For the first ranked ACA only, all participants completed the ERS-ACA scale (Fancourt et al., 2019). Then, participants were asked to select which ACAs from Table 1, if any, they used to do regularly, but had stopped since the COVID-19 pandemic began. As above, up to four “Other” free-text options were available to select. Participants were asked “What prevents you from doing more creative activities? (You can select more than one option)” with the available responses being “I don't feel the need to do more creative activities,” “I don't have time,” “The financial cost,” and a free-text option “Other (please specify).” Finally, participants were asked “Do you generally prefer to do creative activities alone or with others?,” with responses as “Alone,” “With others,” or “I don't have a preference/it depends on the activity.”

In the demographics block, participants were asked to state their age, gender, country of residence, and the cultural background or ethnicity with which they identified. Participants were also asked to respond to two further questions: (1) “How many people currently live in your household (including yourself)?,” with options consisting of numerals ranging 1 to 5, or “6 or more”; and (2) “Are you doing more, less, or the same amount of exercise during the COVID-19 pandemic?” with options being “I am exercising less,” “I am exercising the same amount,” or “I am exercising more.” These variables are henceforth referred to as *Household* and *Exercise*. In the well-being block, participants completed the GAD7, the PHQ9, the “UCLA Before COVID-19” and “UCLA Since COVID-19” scales (see *Measures*).

After completion of the survey participants were able to opt in or out regarding two final aspects. The first aspect was the drawing of a $200 AUD gift card. The second aspect was an invitation to take part in an additional one-on-one interview at a later date. Fifty-six participants agreed to an interview; these responses will be separately analyzed in a forthcoming publication. The survey took ~20 min to complete.

## Results

The survey data were examined according to the ACAs undertaken and ceased during the pandemic and their relationship to well-being and emotion regulation.

### ACAs Undertaken and Ceased During the Pandemic

The ACAs that participants had been undertaking since the onset of the COVID-19 pandemic are listed in Supplementary Table 1, located within the Supplementary Data File. The ACA “Watching films or TV shows” was the most frequently reported, with “Listening to music” and “Cookery or baking” ranked as the second and third most frequent. The ACAs that participants had stopped during the pandemic are reported in Supplementary Table 2. “Dancing” received the highest response (62 responses, being 11.8% of all responses), followed by “Rehearsing/performing play, drama, opera, musical theater” (57 responses, being 10.8% of all responses), “Singing” (47 responses, or 8.9%) and “Photography” (39 responses, or 7.4%). Supplementary Tables 3, 4 report the specific categories that constituted the “Other” ACAs (categorizations made regarding the open-ended responses). These are first listed for the ACAs that were undertaken during the pandemic (Supplementary Table 3), and second for the ACAs that were stopped since the onset of the pandemic (Supplementary Table 4).

### ACAs that Made People “Feel Better”

Supplementary Table 5 reports data concerning participants' rankings for each ACA they had been undertaking in order of effectiveness at making participants “feel better” during the pandemic (where rank 1 equals most effective). The ACAs in Supplementary Table 5 have been sorted by the mean of rank. The most effective ACA at making participants feel better was “Listening to music” (*M* = 3.48), followed by “Other” (*M* = 3.85), “Singing” (*M* = 3.88), “Dancing” (*M* = 4.07), “Gardening…” (*M* = 4.16), “Painting or drawing” (*M* = 4.25), and “Playing a musical instrument” (*M* = 4.44).

### Associations Between Time Spent Undertaking ACAs, Age, Household and Exercise

The sum of the average amount of weekly hours that participants reported spending on each ACA during the pandemic was calculated (*Activity hours*). For total hours spent on all ACAs, *M* = 19.7 and *SD* = 17.1. Following this, descriptive statistics regarding the number of people living in each participant's household were calculated. One hundred and two participants (15.6%) answered that they lived alone (an answer of “1”), 205 participants (31.4%) answered with a Household of “2” people, 143 participants (21.9%) answered with “3,” 137 participants (21%) answered with “4,” 45 participants (6.9%) answered with “5,” and 21 participants (3.2%) answered with “6 or more.” Regarding the amount of exercise that participants had been doing during the pandemic, in comparison to before the pandemic, 327 participants (50.1%) reported that they had been doing less exercise, 161 participants (24.6%) reported that they had been doing the same amount of exercise, and 165 participants (25.3%) reported that they had been doing more exercise.

Following this, a one-way ANOVA was performed, containing Activity hours as the dependent variable, and Age, Exercise, and Household as independent variables. No significant interactions were observed, although significant main effects were observed for Age [*F*_(5, 562)_ = 2.52, *p* = 0.028, $\eta_{p}^{2}$ = 0.022), Exercise [*F*_(2, 562)_ = 5.38, *p* = 0.005, $\eta_{p}^{2}$ = 0.019], and Household [*F*_(5, 562)_ = 2.22, *p* = 0.05, $\eta_{p}^{2}$ = 0.019], although this final main effect is marginally significant (*p*-value not < 0.05). These main effects were followed up with Tukey-corrected *post hoc* tests. Regardless of the significant main effects, all Tukey tests for Age were non-significant at *p* > 0.18, and all Tukey tests for Household were non-significant at *p* > 0.13. That is, we can conclude that there were no significant differences in the amount of overall engagement in ACAs when the sample was split by Age or Household. For Exercise, participants in the “More” category reported spending significantly (*p* = 0.01, *d* = 0.31) more time on ACAs than those in the “The same” category; *M* (*SD*) of activities hours for these categories was 22.2 (20.1) and 16.8 (14.6), respectively, whereas the “Less” category reported *M* (*SD*) of 19.9 (16.4). Both other comparisons concerning Activity hours and Exercise (between the categories “Less” and “The Same,” and “Less” and “More”) were non-significant at *p* = 0.139 and 0.305, respectively.

### Obstacles to Engaging in ACAs During the Pandemic

Responses to the question “What prevents you from doing more creative activities?” are reported in Table 2, although four responses to this question (0.6%) were lost due to a technical fault. It was possible to select multiple answers for this question. Four-hundred and thirty-seven (66.9%) participants selected only one answer; of the single-answer responses “I don't have time” was ranked first (175 responses), “Other” was ranked second (135 responses), “I don't feel the need…” was ranked third (87 responses), and “The financial cost” was ranked last (40 responses). Additionally, 162 participants (24.8%) responded by selecting two answers, 48 participants (7.3%) responded by selecting three of the four answers, and 2 participants (0.3%) selected all four answers. The open-ended “Other” responses were examined and general response themes identified, with multiple themes possible for each participant. The most prevalent theme referred to a lack of motivation, occurring in 70 of the open-ended responses. Following this, references to stress or mental health were observed in 39 open-ended responses, and references to limitations of physical health were observed in 12 open-ended responses. Regarding the question “Do you generally prefer to do creative activities alone or with others?,” for which participants could only select one response, once again four responses to this question (0.6%) were lost due to a technical fault. Three-hundred and fifty-six participants (54.5%) answered “I don't have a preference / it depends on the activity,” 200 participants (30.6%) answered “Alone,” and 93 participants (14.2%) answered “With others.”

**Table 2:** List of responses to the question “What prevents you from doing more creative activities?” Participants selected from one of four answers listed below, and participants were able to select more than one answer.

**Answer (multiple answer strings separated by semicolon)**	**Frequency**
I don't feel the need to do more creative activities	87
I don't have time	175
The financial cost	40
Other (please specify)	135
I don't feel the need to do more creative activities; I don‘t have time	23
I don‘t feel the need to do more creative activities; The financial cost	7
I don‘t feel the need to do more creative activities; Other	18
I don‘t feel the need to do more creative activities; I don‘t have time; Other	5
I don‘t feel the need to do more creative activities; I don‘t have time; The financial cost	12
I don‘t feel the need to do more creative activities; I don‘t have time; The financial cost; Other	2
I don't feel the need to do more creative activities; The financial cost; Other	4
I don't have time; Other	40
I don't have time; The financial cost	45
I don't have time; The financial cost; Other	27
The financial cost; Other	29

### The Use of ACAs and Music Engagement for Emotion Regulation and Well-Being

To provide a general overview of the mental health and well-being of participants, descriptive statistics were calculated for the four well-being scales. And, to understand the general nature of participants' musical engagement during the pandemic, and the emotion regulation strategies that underpinned participants' engagement in the ACA they ranked as most effective at making them “feel better,” descriptive statistics were also calculated for each of the MusEQ and ERS-ACA subscales. These statistics are provided in Table 3.

**Table 3:** Descriptive statistics for the four well-being scales, the six MusEQ subscales, and the four ERS-ACA subscales.

**Scale**	***M (SD)***
**Well-being scales**	
GAD7	13.4 (5.5)
PHQ9	18.4 (6.4)
UCLA Loneliness Before COVID-19	4.0 (1.8)
UCLA Loneliness Since COVID-19	3.3 (2.0)
**MusEQ subscales**	
Daily	3.3 (0.9)
Emotion	3.7 (0.7)
Perform	2.6 (1.0)
Consume	3.2 (0.9)
Respond	3.5 (1.0)
Prefer	3.6 (0.9)
**ERS-ACA subscales**	
General factor	3.8 (0.6)
Avoidance	4.0 (0.7)
Approach	3.6 (0.8)
Self-development	3.9 (0.8)

*GAD 7 ranged from 0 to 21, PHQ9 ranged from 0 to 27, and the UCLA scales ranged from 0 to 6. All remaining scales were rated from 1 to 5. For each well-being scale and ERS-ACA subscale N = 653, whereas for each MusEQ subscale N = 508. This is because the MusEQ questions were only provided to those who reported undertaking at least one music-related ACA during COVID-19*.

To examine any differences in the ways participants engaged with music during the pandemic, as reflected in the MusEQ subscales, a within-subjects ANOVA was performed with these subscales as dependent variables. The ANOVA was significant [*F*_(3.9, 1971.2)_ = 176.35, *p* < 0.001, $\eta_{p}^{2}$ = 0.258], using Greenhouse-Geisser correction as the assumption of sphericity was violated. Šidák-corrected *post hoc* tests are reported in Supplementary Table 6. The *M* values with error bars for the subscales can also be observed in Supplementary Figure 1. The Šidák tests indicated that 13 of the 15 comparisons were significant at *p* < 0.05, with the “Emotion” and “Prefer” subscales being significantly higher than all subscales except for each other. The “Perform” subscale was significantly lower than all other subscales, and the “Consume” subscale, being the second lowest, was significantly lower than all subscales apart from “Daily” (and “Perform”).

To examine any differences in the emotion regulation strategies underpinning participants' engagement in ACAs, the ERS-ACA subscales were used as dependent variables in a second within-subjects ANOVA. The ANOVA was significant [*F*_(3, 1956)_ = 104.31, *p* < 0.001, $\eta_{p}^{2}$ = 0.138], and subsequent Šidák-corrected *post hoc* tests are reported in Supplementary Table 6. The *M* values with error bars for the subscales can also be observed in Supplementary Figure 2. The Šidák tests indicated that the “Avoidance” subscale was significantly higher than all other subscales at *p* < 0.001. The “Self-development” subscale was the second highest and was significantly higher than “General factor” and “Approach” at *p* < 0.001, whereas “Approach” was significantly lower than all other subscales at *p* < 0.001.

### Correlation Analysis

Eighteen variables were subjected to Pearson correlation analysis. Included variables were the four well-being scales, the four ERS-ACA subscales, and the six MusEQ subscales, as well as Activity hours, Age category, Household, and Exercise. Correlation results are reported in Table 4. All four well-being scales showed strong consistency with each other, suggesting that these were appropriately chosen scales for this study. In all cases the relationship was positive and reached significance at *p* < 0.001. Coefficients ranged from 0.308 (GAD7 relating to UCLA Before COVID-19) to 0.769 (GAD7 relating to PHQ9). Activity hours produced a significant, negative correlation with all four well-being scales. Age was also significantly correlated with all four well-being scales, although coefficients were strongest for the GAD7 and PHQ9 scales (*r* = 0.228 and 0.215, respectively). These positive relationships indicate that older participants reported feeling less anxiety, depression, and loneliness. Additionally, as the coefficients were weaker for the UCLA Before COVID-19 scale (*r* = 0.077) than the UCLA Since COVID-19 scale (*r* = 0.130), this suggests that the differences in loneliness between older and younger participants have become more pronounced since the onset of the pandemic.

**Table 4:** Correlation coefficients and significance between the four well-being scales, the four ERS-ACA subscales, the six MusEQ subscales, as well as Activity hours, Age, Household, and Exercise.

**Variables**	**GAD7**	**PHQ9**	**UCLA Before**	**UCLA Since**	**Activity hours**	**Age**	**Household**	**Exercise**	**ERS-ACA General**	**ERS-ACA Avoidance**	**ERS-ACA Approach**	**ERS-ACA Self-devel**.	**MusEQ Daily**	**MusEQ Emotion**	**MusEQ Perform**	**MusEQ Consume**	**MusEQ Respond**
PHQ9	0.769^***^	–															
UCLA Before	0.308^***^	0.359^***^	–														
UCLA Since	0.486^***^	0.508^***^	0.538^***^	–													
Activity hours	−0.091^*^	−0.118^**^	−0.136^***^	−0.125^***^	–												
Age	0.228^***^	0.251^***^	0.077^*^	0.130^***^	−0.004	–											
Household	−0.029	−0.006	0.151^***^	0.071	−0.008	−0.205^***^	–										
Exercise	0.187^***^	0.210^***^	0.057	0.160^***^	0.039	0.071	−0.076	–									
ERS-ACA General	0.042	0.057	0.044	0.000	0.028	0.011	0.067	0.053	–								
ERS-ACA Avoidance	−0.023	−0.016	−0.002	−0.053	0.047	0.013	0.020	−0.003	0.730^***^	–							
ERS-ACA Approach	0.061	0.086^*^	0.062	0.020	−0.001	0.001	0.094^*^	0.076	0.809^***^	0.294^***^	–						
ERS-ACA Self-devel.	0.068	0.067	0.045	0.040	0.022	0.012	0.043	0.053	0.820^***^	0.394^***^	0.603^***^	–					
MusEQ Daily	−0.155^***^	−0.197^***^	−0.061	−0.093^*^	0.226^***^	−0.296^***^	0.092^*^	−0.023	0.128^**^	0.103^*^	0.091^*^	0.105^*^	–				
MusEQ Emotion	−0.224^***^	−0.246^***^	−0.146^***^	−0.189^***^	0.164^***^	−0.182^***^	0.069	0.052	0.214^***^	0.166^***^	0.180^***^	0.150^***^	0.635^***^	–			
MusEQ Perform	−0.119^**^	−0.169^***^	−0.018	0.022	0.110^*^	−0.151^***^	0.094^*^	0.045	0.123^**^	0.005	0.116^**^	0.183^***^	0.481^***^	0.511^***^	–		
MusEQ Consume	−0.110^*^	−0.150^***^	−0.026	−0.019	0.136^**^	−0.120^**^	0.113^*^	0.034	0.095^*^	0.036	0.091^*^	0.100^*^	0.701^***^	0.641^***^	0.689^***^	–	
MusEQ Respond	−0.074	−0.123^**^	0.003	−0.050	0.121^**^	−0.129^**^	0.189^***^	0.037	0.183^***^	0.127^**^	0.138^**^	0.167^***^	0.531^***^	0.560^***^	0.488^***^	0.495^***^	–
MusEQ Prefer	−0.125^**^	−0.132^**^	−0.031	−0.001	0.065	−0.130^**^	−0.017	−0.005	0.031	0.120^**^	−0.060	0.009	0.278^***^	0.418^***^	0.242^***^	0.318^***^	0.176^***^

* *p < 0.05*.

** *p ≤ 0.01*.

*** *p ≤ 0.001*.

*For each correlation N = 653, apart from those concerning the MusEQ scale; the MusEQ scales were only supplied to those selecting musical activities, resulting in N = 508 for these correlations*.

Exercise produced significant correlations with three of the four well-being scales, with the UCLA Before COVID-19 scale being the only one not to reach significance (*r* = 0.057, *p* = 0.145). These positive relationships indicate that participants who exercised more during the pandemic also reported feeling less anxiety, depression, and loneliness. However, we cannot infer the direction or causality of this relationship. While this correlation supports previous findings by Thayer et al. (1994), in which exercise was reported to be the most effective mood-regulation strategy, it is also possible that some or all of the participants in the present study who reported higher levels of well-being were simply motivated to do additional exercise. Household produced a significant relationship with only one well-being scale, being the UCLA Before COVID-19 scale. This positive relationship indicated that those in larger households tended to feel less lonely, although this difference was no longer evident for the UCLA Since COVID-19 scale, suggesting that this effect has reduced due to the pandemic.

Out of 16 possible correlations between the ERS-ACA subscales and the four well-being scales, only one produced significance, and with a weak coefficient (*r* = 0.086, *p* = 0.027). Additionally, out of the 16 possible correlations that could exist between the ERS-ACA scales and the variables Activity hours, Age, Household, and Exercise, only one of these (6.2%) was significant. In contrast to this, out of 24 possible correlations between the four well-being scales and the MusEQ subscales, 14 (58.3%) reached significance. All 14 of these significant correlations outlined a negative relationship, indicating that those with lower Musical Engagement scores tended to produce higher responses (i.e., feeling better) on the four well-being scales. Furthermore, out of the 24 possible correlations that could exist between the MusEQ scales and the variables Activity hours, Age, Household, and Exercise, 15 of these (62.5%) were significant. Five of the six MusEQ scales were positively correlated with Activity hours, and three of the six MusEQ scales were positively correlated with household. All six of the MusEQ scales were negatively correlated with age.

**Figure 1:**
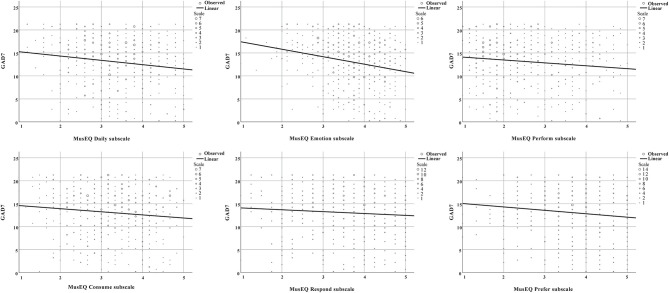
Scatter plot depicting the relationship between the GAD7 scale and the six MusEQ subscales. Data points in the upper half of each plot indicate participants who reported feeling less anxiety, whereas data points in the lower half of each plot indicate participants who reported feeling more anxiety. As per Table 4, a significant negative correlation was observed between the GAD7 scale and each of these subscales.

**Figure 2:**
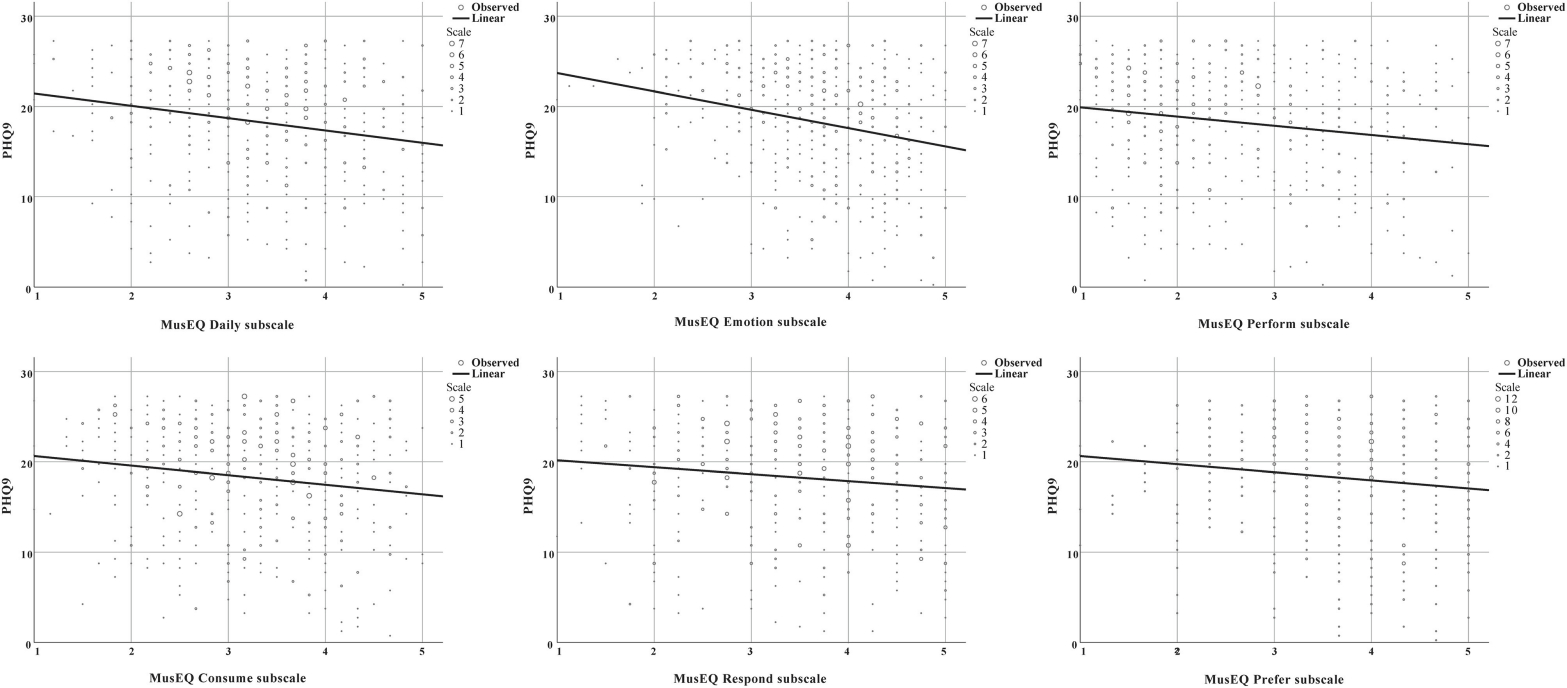
Scatter plot depicting the relationship between the PHQ9 scale and the six MusEQ subscales. Data points in the upper half of each plot indicate participants who reported feeling less depression, whereas data points in the lower half of each plot indicate participants who reported feeling more depression. As per Table 4, a significant negative correlation was observed between the PHQ9 scale and each of these subscales.

The negative correlations between the GAD7 scale and the MusEQ subscales, and also between the PHQ9 scale and the MusEQ subscales, are shown in scatter plots (see Figures 1, 2, respectively). Each figure contains six individual plots, with each plot depicting a MusEQ subscale on the *x*-axis and the well-being scale on the *y*-axis. When examining data in the upper half of each plot (i.e., responses occurring above the halfway point on the depicted well-being scale, referring to participants who reported feeling less anxiety and depression), the data are evenly distributed across the entire *x*-axis. However, when we examine the data in the lower half of each plot, referring to participants who reported feeling more anxiety or depression, five of the six plots for each well-being scale show a negatively skewed distribution. This indicates that participants who reported feeling less anxious and depressed were engaging with music in a variety of ways, specifically ranging from low to high engagement. In contrast, for all of the subscales except for “Perform,” participants who reported feeling more anxious and depressed tended to report high music engagement. This effect is particularly pronounced for the subscales “Daily,” “Emotion,” and “Prefer.”

## Discussion

This study examined the role of artistic creative activities (ACAs) in regulating emotion and supporting the mental health and well-being of Australians during the COVID-19 pandemic. The findings supported our principal hypothesis that ACAs would be used during the pandemic to employ avoidance-based emotion regulation strategies rather than approach- or self-development-based strategies (see *RQ 4* in *Aims*). Fancourt et al. (2019) argued that emotion regulation strategies were linked with positive “healthy” behavioral outcomes regardless of the strategy being employed, stating, “even for ‘avoidance' behaviors when taking part in artistic creative activities, there are no ‘unhealthy' outcomes from the use of these [emotion regulation strategies]” (p. 22; see also Kashdan et al., 2014). Our findings support this statement to a certain extent, since participants completed the ERS-ACA questionnaire regarding the ACA they had identified as being the most effective at making them “feel better.” This suggests that the avoidance strategy that participants deployed via their nominated ACA provided the pathway toward more positive emotional experiences. This argument is also supported by the fact that participants are not likely to have been aware of the emotion regulation choices that were underpinning their engagement in ACAs. This is important, since deliberate and conscious regulatory choices have been a major focus in the field of emotion regulation, even though many emotion regulation choices are likely to be determined implicitly and without deliberate control (Sheppes et al., 2014). It is thus a strength of the current study that we examined emotion regulation strategies during the COVID-19 pandemic via engagement in ACAs. These findings also support previous research which shows that individuals tend toward reappraisal strategies when faced with low-intensity stimuli and distraction or avoidance strategies when faced with high-intensity stimuli such as a global pandemic (Sheppes et al., 2014).

Regarding our first two research questions (*RQ 1* and *RQ 2*), the findings revealed that watching films or TV shows was the most frequently undertaken ACA by participants, followed by listening to music, cookery/baking and reading literature. However, concerning *RQ 3*, “Watching films or TV shows” was ranked as the eighteenth most effective ACA at making participants feel better, with “Listening to music,” “Other” (a conglomeration of mostly arts and crafts activities; see Supplementary Table 3), “Singing,” and “Dancing” ranking as the four most effective ACAs for this purpose. These findings build on previous studies which have suggested that people engage in passive leisure activities significantly more often than activities requiring clear rules, higher energy exertion and greater challenge (“flow” activities) even when they know the latter, more active, activities are more likely to make them happy (Schiffer and Roberts, 2018). This is perhaps because people seem to be generally error-prone in predicting future happiness, as studies in affective forecasting have shown (Gilbert et al., 2002), although it may also be a reflection of the marked increase in clinically significant symptoms of depression in Australia during the pandemic (Fisher et al., 2020), which include low energy levels, feelings of hopelessness and lack of interest in doing things (Kroenke et al., 2001). This, in turn, may help to explain our finding that passive or receptive activities (Davies et al., 2012) accounted for three of the four most frequently undertaken ACAs (these being watching films or TV shows, listening to music, and reading novels, stories, poetry or plays; see Supplementary Table 1).

Our findings also show that music listening was both the ACA most likely to make participants feel better (*RQ 3*), and one that participants could feasibly *do* during the pandemic. This, in conjunction with the results of the within-subjects ANOVA performed on the MusEQ subscales, supported our secondary hypothesis that music would be used during the pandemic for the purposes of emotion regulation. However, the negative correlation between loneliness (both before and during the pandemic) and the MusEQ “Emotion” subscale suggested that people who were less lonely were less likely to engage with music for the purpose of emotion/mood regulation. Furthermore, while dancing and singing were also among the most effective ACAs at making participants feel better (*RQ 3*), they also ranked among the activities that participants were most likely to cease during the pandemic (*RQ 2*), along with rehearsing/performing plays, drama, opera, and musical theater. Notably, three of the top four most effective ACAs at making participants feel better (*RQ 3*) involved music or dance, whereas listening to music was the only music- or dance-related ACA to feature in the top ten most frequently undertaken ACAs during the pandemic (*RQ 1*). This is perhaps because dancing, rehearsing plays (opera, musical theater, etc.) and singing often require significant physical space, and for many people this commodity was at a premium during periods of lockdown. The findings of the current study therefore suggest that music listening is a particularly feasible and efficient ACA for supporting well-being during a pandemic, while also suggesting that singing and dancing are ACAs that may warrant more support during future crises of this kind, given their capacity to make people feel better and the difficulty that participants faced in undertaking these activities. From a mental health and well-being perspective, it may be particularly beneficial for future studies to investigate ways of making singing and dancing more possible and feasible during pandemic crises, as some researchers are already doing (Bohn and Hogue, 2021). Future studies of this kind might also explore possible links between the well-being benefits of engaging in ACAs during a pandemic and profession, which, for reasons of space, could not be explored in the present study. It is reasonable to suggest that professional artists and performers, creative arts and music therapists, and amateur or recreational artists and musicians may engage in ACAs in different ways and for different reasons during periods of enforced lockdown associated with public health crises.

Given that music listening is typically considered a passive or receptive activity (Davies et al., 2012), it is interesting that music listening was the only activity of this type to feature among the ten most effective ACAs at making participants feel better (see Supplementary Table 5), except for “Other,” which itself only includes one passive/receptive activity (see Supplementary Table 3). This suggests that music listening differs in some important well-being-related respects from other passive or receptive ACAs such as reading or watching films. This can perhaps be explained by theories of music listening that frame the activity as active and imaginative rather than as a passive act of perception, such as persona theory (Robinson, 2005; Cochrane, 2010; Peters, 2015). However, this well-being benefit may also be explained by the fact that music listening can be integrated into, or accompany, daily activities such as cooking or doing house chores much better than some other receptive activities such as watching TV/films or reading (DeNora, 2000; Vanstone et al., 2016), which in turn suggests that the well-being benefit may originate in the combination of aesthetic and practical or task-oriented elements. Since music listening ranked as the most effective ACA at making participants feel better during the pandemic, further investigation of the processes by which music listening facilitates well-being benefit is warranted.

Concerning *RQ 5*, the findings also showed that anxious and depressed Australians during the COVID-19 pandemic seem to be turning to music as a coping mechanism or emotional crutch significantly more than people who were not experiencing these mental health issues, or those who were experiencing these issues to a lesser degree. This was also supported by two other findings: a) significant negative correlations between well-being scales and age, indicating that the pandemic had a significantly greater negative impact on the anxiety, depression and loneliness of younger participants than older participants; and b) significant negative correlations between the MusEQ subscales and age, indicating that younger participants showed stronger engagement with music than older participants. This supports prior suggestions that music plays a particularly crucial role in adolescents' development of identity (Tarrant et al., 2002). It also supports the work of Groarke and Hogan (2016), who examined how the adaptive functions of music listening co-function within an enhancement system that supports well-being. These authors observed age differences in the ways that functions of music listening were considered adaptive, with younger participants emphasizing affect regulation and social connection, while older participants emphasized eudaimonic functions such as transcendence and personal growth (p. 769). The finding that younger people were more negatively impacted by the pandemic in terms of anxiety, depression and loneliness than older people also warrants consideration. This could perhaps be a reflection of the destabilizing impact of the pandemic on younger people as they attempt to plan for their future (gaining education and employment, starting families, and so on), although the prevalence of generalized anxiety disorder is expected to decrease with age, partly because emotional control typically increases and anxiety and worry are expressed differently in older adults (Nilsson et al., 2019).

Also concerning *RQ 5*, this study reported significant positive relationships between participants who reported exercising more during the pandemic, and high scores (i.e. feeling better) on three of the four well-being scales. These three scales were specifically for anxiety, depression, and loneliness “since the pandemic.” Furthermore, the fact that this relationship was not present for loneliness “before the pandemic” suggests that the role and importance of exercise on mental health may have increased during the pandemic. However, the causality of these relationships cannot be established. While it is possible that increasing the amount of exercise undertaken during the pandemic had the effect of reducing symptoms of anxiety, depression and loneliness, it is also possible that those who were less impacted by these factors simply felt more motivated to exercise. Indeed, a mixture of both interactions could be present. Similarly, as negative relationships were observed between all four well-being scales and Activity hours, it is possible that an increase in ACA engagement tended to produce small decreases in well-being, or that respondents who felt more anxious, depressed, and lonely were less likely to spend as many hours engaging with ACAs overall; as above, it is not possible to determine the causality of this observed relationship. While no significant relationship was observed between household size and loneliness ratings “since the pandemic,” household produced a significant positive correlation with loneliness ratings “before the pandemic.” This finding suggests that prior to the COVID-19 pandemic, those living in larger households tended to feel less-lonely than those living in smaller households, although this difference has since disappeared. Future studies should examine this relationship in additional detail to pinpoint if certain household sizes provide an optimal boost to well-being during a pandemic, and similarly which household sizes can be expected as most at-risk. Future studies might also explore whether associations exist between demographic data, engagement in ACAs, and other factors that have become associated with life during periods of lockdown such as working from home, home schooling and childcare arrangements.

## Conclusion

We conclude that artistic creative activities, including musical activities, have served as an important resource for facilitating emotion regulation and supporting the mental health and well-being of Australians during the COVID-19 pandemic. Listening to music was the most effective ACA for making participants in this study “feel better,” and while dancing and singing were also ranked as effective, these two ACAs were among the most frequently ceased during the pandemic. We suggest that, where possible, specific ACAs should be prioritized during a pandemic for well-being purposes, and that further research is needed into developing pandemic-safe approaches to dancing and singing, which showed strong emotion-regulatory potential, but which participants found difficult to pursue. Additionally, given its prevalence, further research into what distinguishes music listening from other passive or receptive creative activities (Davies et al., 2012) in terms of well-being benefits may prove beneficial for future crises on a large scale. We also note that younger Australians and those exercising less appear to be the most at risk of experiencing symptoms of anxiety, depression and loneliness. This indicates that specific care needs to be taken in circumstances such as the COVID-19 pandemic to support these at-risk populations. Finally, we argue that these findings indicate that ACAs can form an important and efficient part of Australia's mental health response to pandemic crises.

## Data Availability Statement

The raw data supporting the conclusions of this article will be made available by the authors, without undue reservation.

## Ethics Statement

The studies involving human participants were reviewed and approved by the Human Research Ethics Committee of The University of Melbourne. Written informed consent for participation was not required for this study in accordance with the national legislation and the institutional requirements.

## Author Contributions

FK, JD, MH, and SG developed the research questions and study design and obtained ethics approval. The analyses were undertaken by AC and the findings were discussed by all authors. The first draft of the article was produced by FK and AC with guiding input from JD. Subsequent drafts and revisions also included further substantial intellectual input from MH and SG. All authors helped circulate the survey and recruit participants. The final draft was approved by all authors.

## Conflict of Interest

The authors declare that the research was conducted in the absence of any commercial or financial relationships that could be construed as a potential conflict of interest.

## Publisher's Note

All claims expressed in this article are solely those of the authors and do not necessarily represent those of their affiliated organizations, or those of the publisher, the editors and the reviewers. Any product that may be evaluated in this article, or claim that may be made by its manufacturer, is not guaranteed or endorsed by the publisher.
