# Supplementary material for: The Role of Artistic Creative Activities in Navigating the COVID-19 Pandemic in Australia
Source: Front Psychol. 2021 Aug 25;12:696202. doi: 10.3389/fpsyg.2021.696202 (PMC8423921; doi:10.3389/fpsyg.2021.696202)

The Role of Artistic Creative Activities in Navigating the COVID-19 Pandemic in Australia

doi: 10.3389/fpsyg.2021.696202

Frederic Kiernan[a,*], Anthony Chmiel[a,b], Sandra Garrido[b], Martha Hickey[c,d] & Jane W. Davidson[a]

[a]Melbourne Conservatorium of Music, University of Melbourne, Melbourne, VIC, Australia

[b]The MARCS Institute for Brain, Behaviour, and Development, Western Sydney University, Sydney, NSW, Australia

[c]Department of Obstetrics and Gynaecology, University of Melbourne, Melbourne, VIC, Australia

[d]Royal Women's Hospital, Melbourne, VIC, Australia

*** Correspondence:**
Frederic Kiernan
kiernanf@unimelb.edu.au

Supplementary Material

The following material was collated in this supplementary document to avoid an overly length main document.

This document contains six Supplementary Tables, and two Supplementary Figures. Supplementary Tables and Figures are presented in the order in which they appear in the main document.

Supplementary Table 1.
ACAs undertaken during the pandemic, reported for the entire sample (N = 653). The percentage refers to the number of participants from the sample who reported undertaking this ACA.

Rank	Artistic creative activity (ACA)	Frequency (%) of ACA reported
1	Watching films or TV shows	523 (80.1%)
2	Listening to music	472 (72.3%)
3	Cookery or baking	416 (63.7%)
4	Reading novels, stories, poetry, or plays	398 (60.9%)
5	Gardening/attending indoor plants	332 (50.8%)
6	Painting or drawing	321 (49.2%)
7	Creative writing	239 (36.6%)
8	Viewing/contemplating artworks (books, online…)	239 (36.6%)
9	Textile crafts	229 (35.1%)
10	Photography	203 (31.1%)
11	Singing	186 (28.5%)
12	Playing video games	171 (26.2%)
13	Playing a musical instrument	150 (23%)
14	Dancing	141 (21.6%)
15	Other	104 (15.9%)
16	Making films or videos	100 (15.3%)
17	Creating art/animations on computer	90 (13.8%)
18	Composing music/songs	62 (9.5%)
19	Sculpture	53 (8.1%)
20	Fashion/costume design	49 (7.5%)
21	Make-up artistry	44 (6.7%)
22	Jewellery making	44 (6.7%)
23	Pottery/ceramics	40 (6.1%)
24	Wood crafts (carving, furniture…)	32 (4.9%)
25	Rehearsing/performing play, drama, opera, musical theatre	28 (4.3%)
26	Learning/practising magic tricks/circus skills	23 (3.5%)
27	Calligraphy	22 (3.4%)
	**Total responses**	4710

Supplementary Table 2.
ACAs stopped during the pandemic, reported for the entire sample (N = 653). The percentage is in reference to the total number of reported stopped ACAs.

Rank	Artistic creative activity (ACA)	Frequency (%) reporting ACA stopped
1	Dancing	62 (11.8%)
2	Rehearsing/performing play, drama, opera, musical theatre	57 (10.8%)
3	Singing	47 (8.9%)
4	Photography	39 (7.4%)
5	Watching films or TV shows	36 (6.8%)
6	Painting or drawing	33 (6.3%)
7	Cookery or baking	32 (6.1%)
8	Playing a musical instrument	32 (6.1%)
9	Listening to music	27 (5.1%)
10	Gardening/attending indoor plants	25 (4.7%)
11	Making films or videos	18 (3.4%)
12	Make-up artistry	17 (3.2%)
13	Fashion/costume design	16 (3%)
14	Other	16 (3%)
15	Pottery/ceramics	15 (2.8%)
16	Composing music/songs	10 (1.9%)
17	Creating art/animations on computer	8 (1.5%)
18	Playing video games	8 (1.5%)
19	Wood crafts (carving, furniture…)	8 (1.5%)

20	Sculpture	7 (1.3%)
21	Jewellery making	6 (1.1%)
22	Learning/practising magic tricks/circus skills	6 (1.1%)
23	Calligraphy	1 (0.2%)
24	Creative writing	0 (0%)
25	Reading novels, stories, poetry, or plays	0 (0%)
26	Textile crafts	0 (0%)
27	Viewing/contemplating artworks (books, online…)	0 (0%)
	**Total responses**	526

Supplementary Table 3.

Categorized ACAs from the open-ended descriptions accompanying responses of "Other" regarding ACAs undertaken during the pandemic.

ACAs undertaken during the pandemic (categorization of open-ended "Other" responses)	Count
Other art creation (e.g., resin casting; candle-making; installation art; prop-making…)	19
Puzzles or board/tabletop games	17
Paper crafting	10
Creating or helping new programs/community opportunities for creative activities	10
Interior decoration	9
Listening to podcasts	8
Art with glass, rocks, beads, or mosaics	8
Printmaking	5
Learning something new related to a creative activity (e.g., repairing musical instruments; researching fine art techniques)	4
General activities or playing with children or pets	3
Lego building	3
Learning a new language	2
Building models	2
Making toys for a child (not necessarily wood-crafting)	1
Creating fun activities from numeracy, literacy, art and science school curricula	1
Creating a podcast	1
Making natural healthcare products with essential oils	1
**Total "Other" responses (ACAs undertaken)**	104

Supplementary Table 4.

Categorized ACAs from the open-ended descriptions accompanying responses of "Other" regarding ACAs stopped during the pandemic.

Activities stopped since the onset of the pandemic (categorization of open-ended "Other" responses)	Count
Puzzles or board/tabletop games	4
Creating or helping new programs/community opportunities for creative activities	4
Art with glass, rocks, beads, or mosaics	2
Paper crafting	2
Learning a new language	1
Modelling (model shoots)	1
Listening to podcasts and audiobooks	1
Going to a book club	1
**Total "Other" responses (ACAs stopped)**	16

Supplementary Table 5.
ACAs ranked as the most effective at making participants "feel better" during the pandemic, for the entire sample. Participants gave rankings for each of the ACAs they undertook, from 1 (being the most effective), and increasing in rank as effectiveness decreased. The Overall Rank is based on the Mean of the reported rank of effectiveness for each ACA.

Overall Rank	Artistic creative activity (ACA)	Min. rank	Max. rank	*M* of rank	*SD*	Variance
1	Listening to music	1	13	3.48	2.24	5.02
2	Other	1	13	3.85	2.73	7.46
3	Singing	1	15	3.88	2.77	7.67
4	Dancing	1	14	4.07	2.84	8.08
5	Gardening/attending indoor plants	1	14	4.16	2.75	7.54
6	Painting or drawing	1	14	4.25	2.85	8.13
7	Playing a musical instrument	1	14	4.44	3.03	9.20
8	Textile crafts	1	15	4.45	3.10	9.61
9	Creative writing	1	15	4.85	3.01	9.09
10	Cookery or baking	1	15	4.88	2.98	8.89
11	Composing music/songs	1	16	5.07	3.21	10.33
12	Reading novels, stories, poetry, or play	1	18	5.17	2.94	8.64
13	Pottery or ceramics	1	14	5.30	3.61	13.06
14	Fashion or costume design	1	16	5.30	3.20	10.21
15	Wood crafts (carving, furniture…)	1	14	5.50	3.04	9.25
16	Sculpture	1	12	5.60	3.28	10.79
17	Rehearsing/performing play, drama, opera, musical theatre	1	17	5.62	4.23	17.85
18	Watching films or TV shows	1	16	5.65	2.95	8.72
19	Playing video games	1	17	6.07	3.56	12.65

20	Learning/practising magic tricks/circus skills	1	14	6.27	4.45	19.83
21	Making films or videos	1	16	6.29	3.29	10.83
22	Calligraphy	1	12	6.30	3.33	11.08
23	Creating art/animations on computer	1	14	6.32	3.33	11.11
24	Photography	1	16	6.41	3.46	11.97
25	Jewellery making	1	13	7.13	3.2	10.25
26	Viewing/contemplating artworks (books, online…)	2	16	7.21	2.78	7.7
27	Make-up artistry	2	16	7.69	3.74	13.98

Supplementary Table 6.

Significance and effect size (Cohen's d) for Šidák-corrected post hoc tests between the MusEQ subscales and also between the ERS-ACA subscales.

**MusEQ subscales compared**			***p***	***d***
Daily	–	Emotion†	<.001	0.43
Daily†	–	Perform	<.001	0.75
Daily	–	Consume	.089	0.10
Daily	–	Respond†	.004	0.16
Daily	–	Prefer†	<.001	0.35
Emotion†	–	Perform	<.001	1.20
Emotion†	–	Consume	<.001	0.54
Emotion†	–	Respond	<.001	0.23
Emotion	–	Prefer	>.999	0.03
Perform	–	Consume†	<.001	0.67
Perform	–	Respond†	<.001	0.87
Perform	–	Prefer†	<.001	1.08
Consume	–	Respond†	<.001	0.26
Consume	–	Prefer†	<.001	0.45
Respond	–	Prefer†	.028	0.17
**ERS-ACA subscales compared**			***p***	***d***
General factor	–	Avoidance†	<.001	0.30
General factor†	–	Approach	<.001	0.37
General factor	–	Self-development	.122	0.06
Avoidance†	–	Approach	<.001	0.61
Avoidance†	–	Self-development	<.001	0.21
Approach	–	Self-development†	<.001	0.38

Note. † is included for significant comparisons, to denote which subscale was rated higher.

Supplementary Figure 1.

Mean ratings for the six MusEQ subscales. The 35 items were rated from 1 ("Not at all") to 5 ("Very much"). Error bars = ±1 SE.

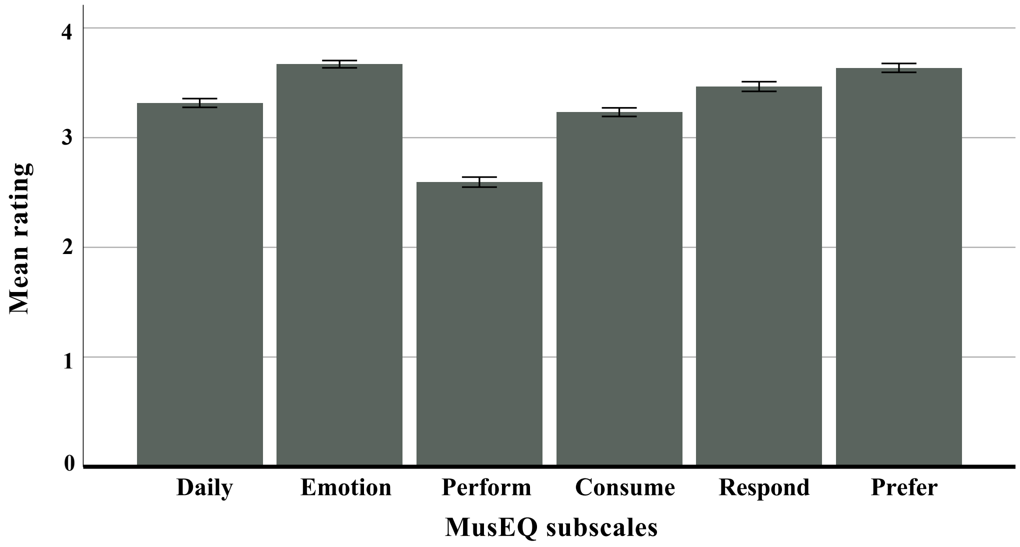

Supplementary Figure 2.

Mean ratings for the four ERS-ACA subscales. The 18 items were rated from 1 ("Strongly disagree") to 5 ("Strongly agree"). The "General factor" subscale is the overall mean value of all 18 items, whereas the Avoidance, Approach, and Self-development subscales are each the mean value of 7 items, 6 items, and 5 items respectively. Error bars = ±1 SE.

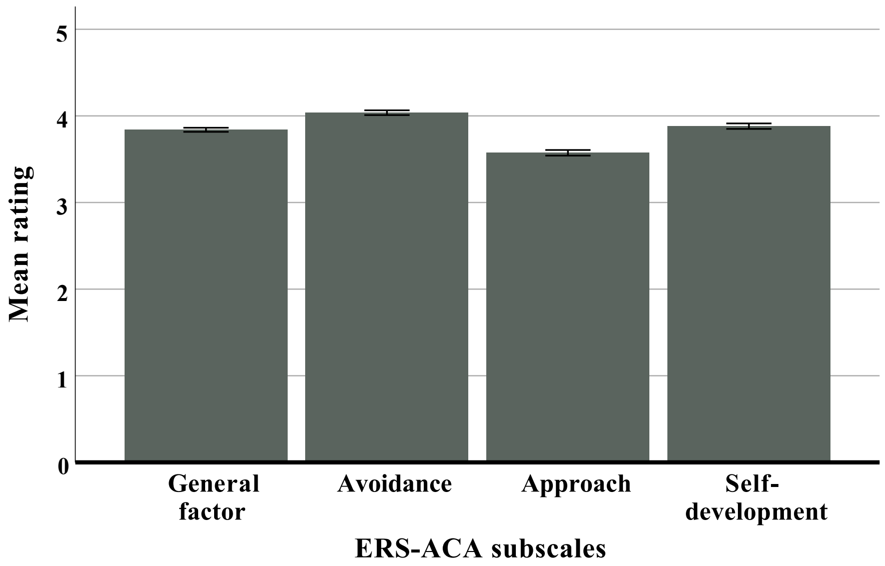

Supplement: Supplementary file 1 [file Data_Sheet_1.pdf]
